# Pseudomonas orientalis F9 Pyoverdine, Safracin, and Phenazine Mutants Remain Effective Antagonists against Erwinia amylovora in Apple Flowers

**DOI:** 10.1128/AEM.02620-19

**Published:** 2020-04-01

**Authors:** Amanda Santos Kron, Veronika Zengerer, Marco Bieri, Vera Dreyfuss, Tanja Sostizzo, Michael Schmid, Matthias Lutz, Mitja N. P. Remus-Emsermann, Cosima Pelludat

**Affiliations:** a Agroscope, Research Division Plant Protection, Wädenswil, Switzerland; b Agroscope, Research Division Phytosanitary Service, Wädenswil, Switzerland; c Genexa AG, Zürich, Switzerland; d Agroscope, Research Division Extension Vegetable Growth, Wädenswil, Switzerland; e School of Biological Sciences, University of Canterbury, Christchurch, New Zealand; f Biomolecular Interaction Centre, University of Canterbury, Christchurch, New Zealand; Nanjing Agricultural University

**Keywords:** *Erwinia amylovora*, *Malus domestica*, biocontrol, cress assay, detached flower assay, fire blight, safracin, siderophore, transposon mutagenesis

## Abstract

The recently characterized strain Pseudomonas orientalis F9, an isolate from apple flowers in a Swiss orchard, exhibits antagonistic traits against phytopathogens. At high colonization densities, it exhibits phytotoxicity against apple flowers. P. orientalis F9 harbors biosynthesis genes for the siderophore pyoverdine as well as for the antibiotics safracin and phenazine. To elucidate the role of the three compounds in biocontrol, we screened a large random knockout library of P. orientalis F9 strains for lack of pyoverdine production or *in vitro* antagonism. Transposon mutants that lacked the ability for fluorescence carried transposons in pyoverdine production genes. Mutants unable to antagonize Erwinia amylovora in an *in vitro* double-layer assay carried transposon insertions in the safracin gene cluster. As no phenazine transposon mutant could be identified using the chosen selection criteria, we constructed a site-directed deletion mutant. Pyoverdine-, safracin-, and phenazine mutants were tested for their abilities to counteract the fire blight pathogen Erwinia amylovora *ex vivo* on apple flowers or the soilborne pathogen Pythium ultimum *in vivo* in a soil microcosm. In contrast to some *in vitro* assays, *ex vivo* and *in vivo* assays did not reveal significant differences between parental and mutant strains in their antagonistic activities. This suggests that, *ex vivo* and *in vivo*, other factors, such as competition for resources or space, are more important than the tested antibiotics or pyoverdine for successful antagonism of P. orientalis F9 against phytopathogens in the performed assays.

**IMPORTANCE** Pseudomonas orientalis F9 is an antagonist of the economically important phytopathogen Erwinia amylovora, the causal agent of fire blight in *pomme* fruit. On King’s B medium, P. orientalis F9 produces a pyoverdine siderophore and the antibiotic safracin. P. orientalis F9 transposon mutants lacking these factors fail to antagonize E. amylovora, depending on the *in vitro* assay. On isolated flowers and in soil microcosms, however, pyoverdine, safracin, and phenazine mutants control phytopathogens as clearly as their parental strains.

## Abstract (precis)

Pseudomonas orientalis F9 is an antagonist of the economically important phytopathogen Erwinia amylovora, the causal agent of fire blight in *pomme* fruit. On King’s B medium, P. orientalis F9 produces a pyoverdine siderophore and the antibiotic safracin. P. orientalis F9 transposon mutants lacking these factors fail to antagonize E. amylovora, depending on the *in vitro* assay. On isolated flowers and in soil microcosms, however, pyoverdine, safracin, and phenazine mutants control phytopathogens as clearly as their parental strains.

## INTRODUCTION

The ongoing concerns over the application of pesticides in plant protection intensify the need for management strategies that include the use of antagonists. For their optimal selection and use in agriculture, an understanding of their mode of action is mandatory. Recently, the antagonistic activity of Pseudomonas orientalis F9, an isolate obtained from apple flowers in a Swiss orchard, was tested against several phytopathogenic strains *in vitro* and *ex vivo* (1). P. orientalis F9 induced *in vitro* a growth deficiency in the fire blight pathogen Erwinia amylovora CFBP1430^Rif^ (E. amylovora^Rif^). The fire blight pathogen affects apple, pear, and quince and is a major threat to fruit production (2). In 2016, the most effective control in fire blight management, the antibiotic streptomycin, was banned from field applications in Switzerland due to the possible rise and spread of resistant pathogens (3, 4). Antagonists are a desirable alternative to antibiotics if their efficacy in reduction of blossom infection is comparable to that of the most effective controls; regardless of the environmental conditions. However, the reduction in blossom infection with biological treatments in experiments conducted between 2001 and 2007 ranged from 9.1 to 36.1% while values for the control with streptomycin ranged from 59 to 67.3% (5). In addition to E. amylovora, P. orientalis F9 also reduced growth of plant pathogens belonging to pathovars of Pseudomonas syringae (P. syringae pv. syringae ACW, P. syringae pv. actinidiae ICMP 9617, and P. syringae pv. persicae NCPPB 2254) in an *in vitro* double-layer assay. However, *ex vivo* on apple flowers, P. orientalis F9 revealed phytotoxic properties when inoculated at a high dose (1).

The genome of P. orientalis F9 contains genes for the synthesis of pyoverdine (siderophore) and for the synthesis of the antibiotics safracin and phenazine-1-carboxylic acid. Phenazines have an antibiotic activity against bacteria, fungi, and eukaryotes. They are known to successfully suppress soilborne pathogens (6–8). Indeed, when tested in a cress assay, F9 revealed antagonistic activity against the soilborne pathogen Pythium ultimum (1). Safracin belongs to the tetrahydroisoquinoline (THIQ) alkaloids, known for their broad-spectrum antibacterial activities (9) and especially strong antitumor activities. The biosynthetic gene clusters of six THIQ antibiotics have been characterized, including ET-743 that has been commercialized as anticancer drug (10). Siderophores are iron chelators that bacteria produce to scavenge iron in iron-deficient environments. Pyoverdines are the fluorescent pigments produced by *Pseudomonas* species and their primary siderophore (11, 12). Siderophores and antibiotics have both been shown to be involved in antagonistic activities against plant pathogens (11, 13). In the present study, we analyzed the antagonistic traits of P. orientalis F9 with regard to the siderophore pyoverdine and the antibiotics safracin and phenazine for further understanding and selection of appropriate antagonists.

Transposon mutagenesis and subsequent selection criteria (fluorescence on iron-limited King’s B [KB] medium and halo induction of E. amylovora in a double-layer assay) led to the identification of pyoverdine and safracin mutants. No phenazine transposon mutant was selected. To exclude the possibility of phenazine as a major player in the antagonistic traits of P. orientalis F9, a site-directed mutant of the phenazine cluster of F9 was constructed.

P. orientalis F9 transposon and site-directed mutants were analyzed for their antagonistic activity in order to correlate phenotypic traits of the mutants and their abilities to counteract phytopathogens.

## RESULTS

### Selected transposon mutants and site of transposon integration.

**FIG 1:**
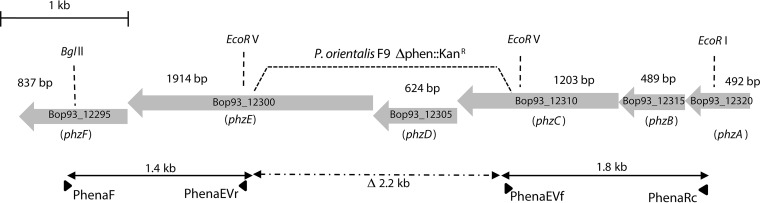
Schematic representation of the phenazine gene cluster in P. orientalis F9. Large gray arrows indicate coding sequences. Small black horizontal arrows show positions and directions of primers used for site-directed mutagenesis. The PCR-generated fragments were cut at naturally occurring EcoRV restriction sites for insertion of the kanamycin resistance cassette, leading to deletion of a 2.2-kb fragment of the phenazine cluster in P. orientalis F9 (Δphen::Kan^r^). Genes were assigned with their corresponding accession numbers from the sequenced P. orientalis F9 genome and correspond to the six genes *phzABCDEF* of the phenazine cluster (from right to left).

The genome of P. orientalis F9 (GenBank accession number CP018049.1) is 5.99 Mbp, with an average GC content of 60.4% and no plasmids. Genome analysis revealed that the genome carries a safracin production cluster, a phenazine-1-carboxylate operon (*phzABCDEFG*) (Fig. 1), and pyoverdine synthesis genes (1).

**FIG 2:**
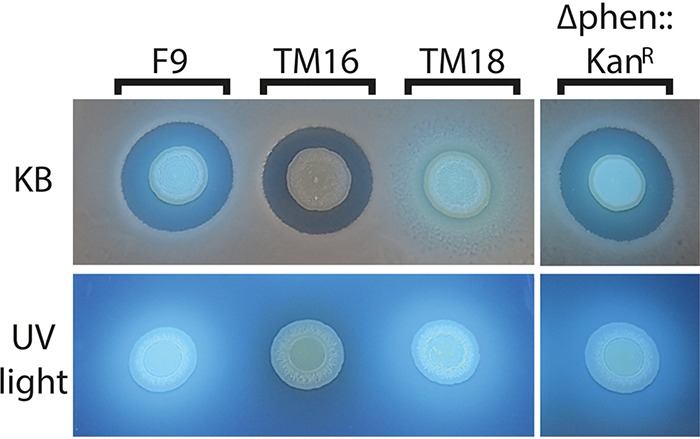
Double-layer assay with P. orientalis F9 (F/H), transposon mutants TM16 (nF/H) and TM18 (F/nH), and phenazine mutant P. orientalis F9 (Δphen::Kan^r^) (F/H) pipetted onto E. amylovora CFBP1430 seeded in KB overlay agar. Presence and absence of E. amylovora inhibition zones are visualized in the KB medium double-layer assay; UV light reveals the presence or absence of the fluorescence that is indicative for siderophore production. nF/H, no fluorescence/halo induction; F/nH, fluorescence/no halo induction; F/H, fluorescence/halo induction.

P. orientalis F9 was subjected to random insertion transposon mutagenesis in order to mutagenize potential antibiotics and siderophore genes. F9 transposon mutants were screened using a double-layer technique on KB agar seeded with E. amylovora^Rif^. Mutants were selected based on their inability to cause growth inhibition halos of the pathogen or on the absence of fluorescence when they were grown on KB plates. The transposon mutants were grouped into two phenotypic groups: no fluorescence/halo induction (nF/H) and fluorescence/no halo induction (F/nH) (Fig. 2).

The insertion site of the transposon on the P. orientalis F9 chromosome was determined for 15 mutants of group 1 (nF/H) and 4 mutants of group 2 (F/nH) using arbitrary PCR. All nF/H mutants carried the transposons within the 19 genes predicted to be part of the pyoverdine synthesis cluster, and F/nH mutants carried the transposons within the 10 genes with high similarity to the safracin cluster in Pseudomonas fluorescens A2-2 (9).

**FIG 3:**
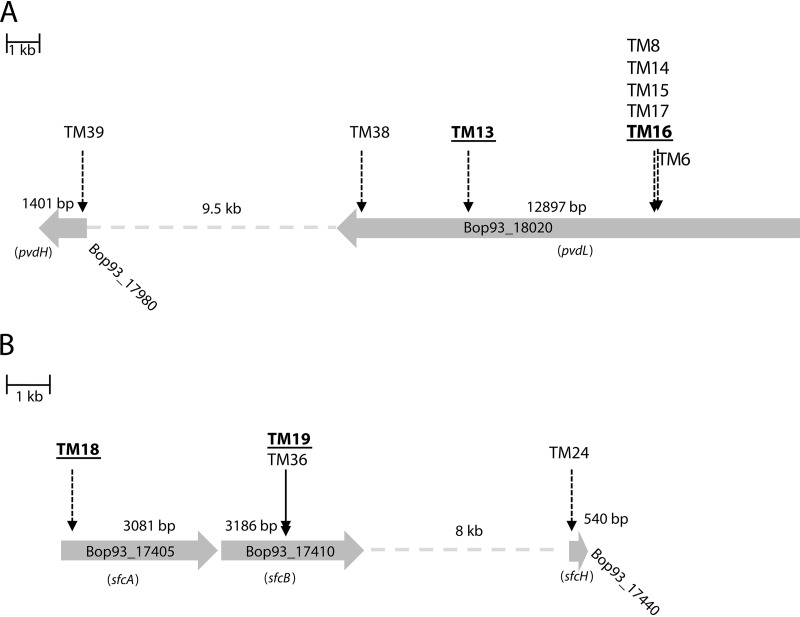
Schematic representation of transposon location for each defined phenotype group. Coding sequences are indicated by large horizontal arrows which show the directions of transcription. Black vertical arrows show the integration sites of transposons. Dashed lines represent intermediate DNA regions (length in kilobases). Genes were assigned to the annotated P. orientalis F9 genome with their corresponding Bop number. (A) Transposon location of fluorescence-negative but halo-inducing mutants (nF/H) in genes Bop93_17980 and Bop93_18020. Homology study allocated the two genes to the pyoverdine gene cluster. TM16 and TM13 were used in further analysis. (B) Transposon location of fluorescent but not halo-inducing mutants (F/nH). Insertion occurred in three different genes of the safracin cluster. TM18 and TM19 were used in further analysis.

Nine of the nF/H selected transposon mutants carried their transposons in Bop93_18020, which encodes a protein with 91% amino acid identity to PvdL, a pyoverdine chromophore precursor synthetase of the fluorescent bacterium Pseudomonas synxantha BG33R. Transposon mutants TM8, TM14, TM15, TM16, and TM17 shared the same insertion site in Bop93_18020, possibly due to clonal origin, while TM13 and TM38 carried their transposons closer to the 3′ end of the gene (Fig. 3A). Mutant TM10 (nF/H; data not shown) carries the transposon in BOP93_10425, a gene with homology to *pvdF* encoding the pyoverdine synthetase of Pseudomonas protegens Pf-5 (PFL_4090).

TM18 (F/nH) carries an insertion in Bop93_17405. Bop93_17405 encodes a protein that is more than 78% amino acid identical to SfcA, which is part of the safracin cluster in P. fluorescens ATCC 13525. TM19 (F/nH) is a transposon mutant of Bop93_17410 which is also part of the safracin cluster (*sfcB*) (Fig. 3B; Table 1). An additional F/nH mutant was detected with the transposon insertion in Bop93_17440 which encodes a protein that has identity to SfcH (85%). For subsequent analyses in various assays, TM16 and TM18 as representative of each phenotypic group were selected.

**TABLE 1:** Strains and plasmids used in this study

Strain or plasmid	Notes^*a*^	Reference or source
Strains		
P. orientalis F9 strains		
Wild type	Isolated from Malus domestica flower, canton Zurich, Switzerland, in 2014; F/H	1
TM10	Transposon mutant of F9; nF/H	This study
TM13	Transposon mutant of F9; nF/H	This study
TM16	Transposon mutant of F9; nF/H	This study
TM18	Transposon mutant of F9; F/nH	This study
TM19	Transposon mutant of F9; F/nH	This study
Δphen::Kan^r^ strain	Phenazine mutant F9 with deletion of *phzCDE*; F/H	This study
E. amylovora CFBP1430^Rif^	Spontaneous rifampicin-mutant of E. amylovora CFBP1430	1
P. vagans C9-1	E. amylovora antagonist	16
P. syringae pv. persicae NCPPB 2254	Causal agent of bacterial die-back in peach, nectarine, Japanese plum	CFBP^*b*^
P. syringae pv. actinidiae ICMP 9617	Causal agent of bacterial canker of kiwifruit	CFBP
P. syringae pv. syringae ACW	Causal agent of bacterial canker of *pomme* and stone fruit	1
P. protegens CHA0	Model organism in biological control of soilborne pathogens	51
E. coli S17-1 λpir	*pir*^+^ *tra*^+^, Sm^r^	52
E. coli SM10 λpir(pJA1)	Contains the suicide vector pJA1; *pir*^+^ *tra*^+^, Kan^r^	52
P. ultimum	Soilborne phytopathogen	51
Plasmids		
pKAS32	Cloning vector with *rpsL* gene, Amp^r^	42
pSB315	Containing kanamycin cassette without transcriptional terminator, Amp^r^ Kan^r^	43
pJA1	Containing transposon, Kan^r^	37

a F/H, fluorescence/halo induction; nF/H, no fluorescence/halo induction; F/nH, fluorescence/no halo induction.

b CFBP, Collection Française de Bactéries associées aux Plantes.

No transposon mutant with an insertion in the phenazine gene cluster was identified. Thus, site-directed mutagenesis was performed, leading to a 2.2-kb deletion in the phenazine operon in P. orientalis F9 (here, the Δphen::Kan^r^ strain). The mutant strain showed fluorescence when grown on KB plates and E. amylovora halo induction in the double-layer assay (F/H phenotype) (Fig. 1 and 2; Table 1).

### Growth of P. orientalis F9 and corresponding mutants in PSBM and KB medium.

**FIG 4:**
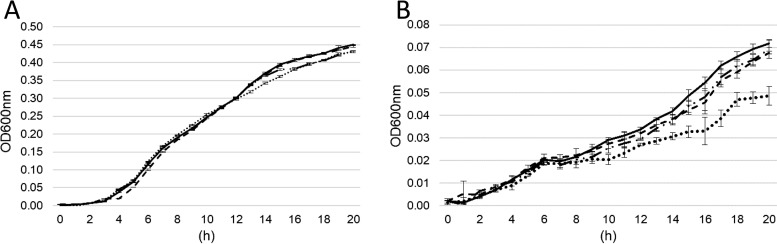
Growth curve of P. orientalis F9 (black line), P. orientalis F9 (Δphen::Kan^r^) (broken line), nF/H mutant TM16 (dotted line), and F/nH mutant TM18 (dotted broken line) in KB medium (A) and PSBM (B). Error bars represent standard deviations.

The growth of P. orientalis F9 and its mutants was evaluated in partial stigma-based medium (PSBM) (14), which mimics the nutrient composition on the stigma, and in iron-limited KB medium. In PSBM, TM16 showed less growth than TM18, F9 (Δphen::Kan^r^), or the parental strain. In KB medium all strains grew equally well (Fig. 4). When tested on siderophore indication agar (chrome azurol S [CAS] agar), all strains, including TM16, produced halos of the same size (see Fig. S1 in the supplemental material). The CAS phenotype and growth of TM16 in KB medium indicate an additional siderophore system present in P. orientalis F9. However, genome analysis of F9 failed to identify additional secondary siderophores (yersiniabactin, pyochelin, achromobactin, PDTC [pyridine-2,6-bis(monothiocarboxylic acid)], or thioquinolobactin).

### Ability of F9 transposon mutants to inhibit growth of phytopathogens and antagonists *in vitro*.

**FIG 5:**
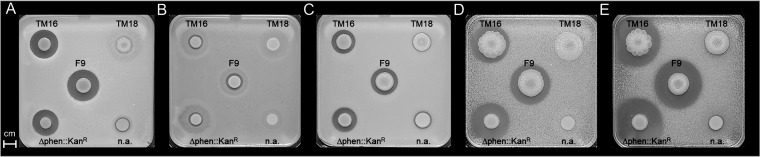
Double-layer assay of P. orientalis F9 and corresponding mutant strains and their impact on the growth of plant pathogens and antagonists seeded in a KB medium overlay. (A) E. amylovora CFBP1430. (B) P. vagans C9-1 (E. amylovora antagonist). (C) P. syringae pv. syringae ACW. (D) P. syringae pv. actinidiae ICMP 9617. (E) P. syringae pv. persicae NCPPB 2254. The following strains were spotted on top: P. orientalis F9, P. orientalis F9 (Δphen::Kan^r^), nF/H mutant TM16, and F/nH mutant TM18, as indicated. n.a., not applicable.

A previous study demonstrated that P. orientalis F9 is capable of inhibiting the growth of bacterial phytopathogens and antagonists *in vitro* (1). Strains E. amylovora^Rif^, the E. amylovora antagonist Pantoea vagans C9-1, P. syringae pv. syringae ACW, P. syringae pv. actinidiae ICMP 9617, and P. syringae pv. persicae NCPPB 2254 (15–17) were poured into the top layer of a double-layer assay. The assay revealed that the nonfluorescent TM16 mutant (nF/H) and P. orientalis F9 (Δphen::Kan^r^) (F/H) induced a growth deficiency similar to that of the parental strain P. orientalis F9. The fluorescent but safracin-negative mutant TM18 (F/nH), on the other hand, had no impact on the growth of the tested strains (Fig. 5).

### Ability of F9 transposon mutants to inhibit growth of P. ultimum in a cress assay.

**FIG 6:**
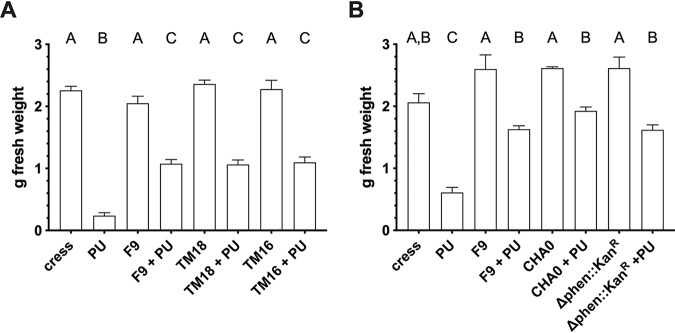
(A) Cress biomass after treatment with P. ultimum (PU) and/or P. orientalis F9 and corresponding transposon mutants TM18 and TM16. (B) Cress biomass after treatment with P. ultimum (PU) and/or P. orientalis F9, P. orientalis F9 (Δphen::Kan^r^), and known antagonist P. protegens CHA0, as indicated. Cress biomass was harvested at 7 days postinoculation. Error bars depict the standard errors of the means. Different letters depict significant differences between measurements (one-way analysis of variance and multiple-comparison test with Tukey’s correction, *P* < 0.0001).

A cress assay was performed with P. orientalis F9 and mutant strains. As shown previously (1), P. orientalis F9 was similarly effective as the established antagonist P. protegens CHA0 (Fig. 6B) when cress was coinoculated with the soilborne pathogen P. ultimum in soil. P. orientalis F9 and its mutants were applied onto soil containing P. ultimum and cress. Transposon mutants TM16, TM18, and P. orientalis F9 (Δphen::Kan^r^) exhibited no statistically significant difference compared to results with the parental strain (Fig. 6A and B).

### Growth inhibition of E. amylovora^Rif^ in an *in vitro* competition assay using stigma-based medium.

**FIG 7:**
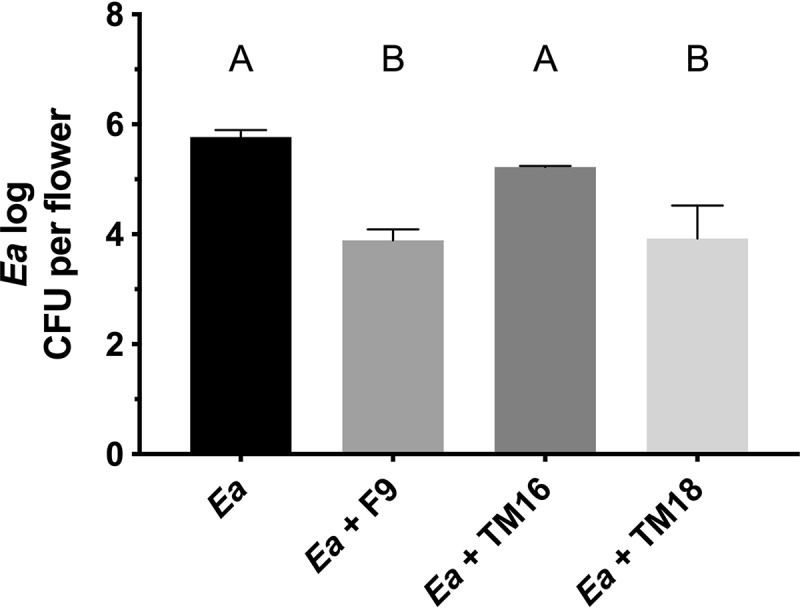
*In vitro* competition assay of P. orientalis F9 and transposon mutants TM16 (nF/H) and TM18 (F/nH), cocultivated with E. amylovora^Rif^ (*Ea*) in PSBM at 26°C. E. amylovora^Rif^ CFU counts were determined after 3 days of incubation. Error bars represent standard deviations of the means. Different letters depict significant differences between measurements (one-way analysis of variance and multiple-comparison test with Tukey’s correction, *P* < 0.05).

P. orientalis F9 originates from apple flowers, and despite its phytopathogenic traits in the flower, the strain also significantly reduces E. amylovora^Rif^ CFU counts on flowers. To elucidate whether or not siderophore and safracin deficiency has an impact on antagonistic activity *in vitro*, a PSBM competition assay was performed. PSBM mimics nutrients present on the stigma. E. amylovora^Rif^ was coinoculated with P. orientalis F9 or transposon mutants. E. amylovora^Rif^ CFU counts were determined after 3 and 4 days of incubation. In contrast to results from the double-layer assay with KB medium, the nonfluorescent mutant TM16 revealed reduced antagonistic activity against the fire blight pathogen, which is in accordance with PSBM growth curve results (Fig. 4B), in which TM16 showed reduced growth. There was no significant difference in the CFU counts of E. amylovora^Rif^ coinoculated with P. orientalis F9 or the safracin mutant TM18 (Fig. 7).

### Inhibition of E. amylovora^Rif^ by P. orientalis F9 mutants in a detached-flower assay.

**FIG 8:**
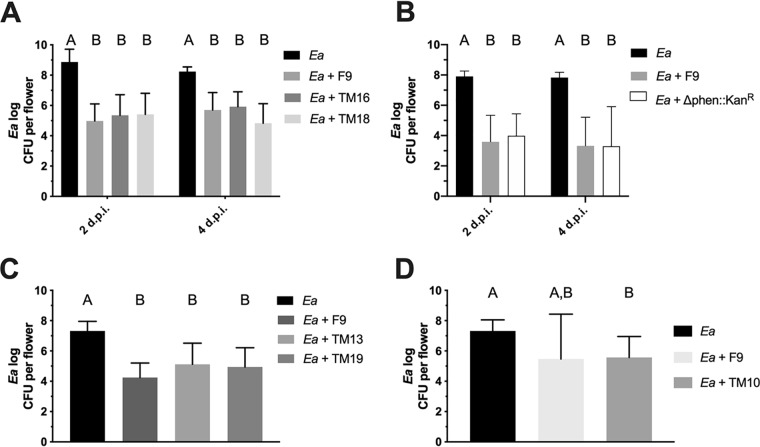
Antagonistic activity of P. orientalis F9 and mutants against E. amylovora^Rif^ (*Ea*) in apple flowers. (A and B) Recovered CFU counts (log) of E. amylovora^Rif^ control and E. amylovora^Rif^ coinoculation with P. orientalis F9, nF/H mutant TM16), and F/nH mutant TM18 or P. orientalis F9 (Δphen::Kan^r^) after 2 and 4 days postinfection. (C and D) Antagonistic activity of P. orientalis F9 alternative mutants TM13 (nF/H) and TM19 (F/nH) and TM10 (nF/H), as indicated, at 5 days postinfection. Error bars represent standard deviations of the means. Different letters depict significant differences between measurements (two-way analysis of variance and multiple-comparison test with Tukey’s correction, *P* < 0.005).

Flowers are the primary location in which E. amylovora replicates and subsequently invades the plant host tissue. Thus, growth reduction of E. amylovora in the flowers is one of the most important tasks of fire blight management. To evaluate the impact of pyoverdine, phenazine, and safracin on the antagonistic activity of P. orientalis F9 against E. amylovora^Rif^ in the apple flower, a detached-flower assay was performed. P. orientalis F9 and transposon mutants TM16 (nF/H) and TM18 (F/nH) as well as the deletion mutant P. orientalis F9 (Δphen::Kan^r^) were coinoculated with E. amylovora^Rif^ onto the hypanthium of apple flowers. After 2 and 4 days of incubation, E. amylovora^Rif^ was reisolated from the apple flowers, and the CFU counts were determined on selective medium. No significant difference in the determined CFU counts could be detected (Fig. 8A and B). This was also true for alternative transposon mutants TM13 (nF/H), TM19 (F/nH), and TM10 (nF/H) (Fig. 8C and D).

Thus, in the detached-apple flower assay, the parental P. orientalis F9 strain and all tested mutants had the same antagonistic activities against the fire blight pathogen. In addition, when P. orientalis F9, TM16, and TM18 and P. orientalis F9 (Δphen::Kan^r^) were inoculated solely with a high inoculum in apple flowers, all strains revealed phytotoxic effects (Fig. S2). Thus, in the performed detached-flower assay, neither the siderophore nor the tested antibiotics are major participants in antagonistic traits or necrosis of apple flowers.

## DISCUSSION

P. orientalis F9 has been shown to reduce growth of phytopathogenic microorganisms *in vitro* and *ex vivo* (1). We analyzed the impact of the strain’s antibiotics safracin and phenazine as well as its siderophore pyoverdine on its antagonistic traits.

The main siderophores produced under iron-limiting conditions by fluorescent pseudomonads, including P. orientalis F9, are pyoverdines. Thus, transposon mutants of P. orientalis F9 were selected according to their loss of fluorescence on iron-limited KB plates, indicative of the absence of pyoverdine. Additionally, mutants that exhibited loss of growth halo induction were selected using a double-layer assay with E. amylovora^Rif^ seeded as an indicator strain in a top layer of KB medium. Halo-inducing negative mutants were shown to carry transposon insertions within genes of the safracin operon (Fig. 3B, TM18 and TM19). TM16, TM13, and TM10 represented the nonfluorescent, but halo-forming phenotype, with the transposon inserted in the pyoverdine synthesis genes (Fig. 3A). As no transposon insertion in the annotated phenazine synthesis genes of P. orientalis F9 could be identified, site-directed mutagenesis was performed, resulting in P. orientalis F9 (Δphen::Kan^r^) (Fig. 1).

In the double-layer assay using KB medium, nonfluorescent mutant TM16 revealed growth reduction not only of E. amylovora^Rif^ but also of P. syringae pathovars and the E. amylovora antagonist P. vagans C9-1, similar to results with the wild type. The same was true for P. orientalis F9 (Δphen::Kan^r^) (Fig. 5). In contrast, the fluorescent mutant TM18 with the transposon positioned in the *sfcA* homolog of F9 was incapable of inducing a halo in any of the seeded strains (Fig. 5). This indicates that in this assay the production of safracin is sufficient to negatively impact growth of the tested strains. Therefore, the antagonistic activity of P. orientalis F9 in the double-layer assay can be attributed to the production of safracin but not to that of pyoverdine or phenazine. In contrast, when strains were tested in an *in vitro* competition assay in PSBM, which mimics the nutrient composition of the stigma (14), only the siderophore-negative mutants TM16 (Fig. 7) and TM13 (data not shown) failed to reduce the CFU count of E. amylovora^Rif^. This is in correspondence with the weaker growth of TM16 in PSBM than that of the other strains (Fig. 4).

When tested in the *in vivo* cress assay and in the *ex vivo* detached apple flower assay, the P. orientalis F9 mutants have the same antagonistic activity against E. amylovora^Rif^ and the model soil pathogen P. ultimum as the parental strain (Fig. 6 and 8). The recovered CFU counts of E. amylovora^Rif^ after coinoculation into apple flowers with P. orientalis F9, corresponding transposon mutants TM16 and TM18, alternative transposon mutants TM13, TM10, and TM19, or P. orientalis F9 (Δphen::Kan^r^) did not differ significantly (Fig. 8). The same was true for the cress biomass defined after coinoculation of the pathogen with parental or mutant strains (Fig. 6). E. amylovora produces the hydroxamate siderophore desferrioxamine E (DFO E) (18–20). The importance of this system for the pathogenicity of E. amylovora has been demonstrated using mutants deficient in siderophore synthesis or uptake (21). The lack of pyoverdine production in TM16 and the subsequent reduced competition of the mutant strain for iron in the apple flower were expected to impact its ability to counteract E. amylovora. Thus, it was unexpected that TM16 was still able to significantly reduce E. amylovora^Rif^ in the detached-flower assay. When poured on CAS agar, TM16 produced a halo (indicative for siderophore synthesis) (see Fig. S1 in the supplemental material) equal in size to that of parental strain F9. Genome analysis could not reveal additional synthesis genes for secondary siderophores such as yersiniabactin, pyochelin, achromobactin, PDTC, or thioquinolobactin. Either F9 carries an unidentified system, or the CAS assay interferes with alternative iron chelating agents, e.g., citrate that triggered the CAS signal in case of Bradyrhizobium japonicum and enabled the strain to take up iron (22). In case of the marine pathogen Photobacterium damselae subsp. *damselae*, a mutant lacking the citrate synthase (GltA) showed almost no reaction in the CAS test (23). The P. orientalis F9 chromosome encodes a protein with 72.5% identity. In addition, phenazine-1-carboxylic acid production also has an impact on iron availability; e.g., strains producing the antibiotic can have an enhancing effect on the reactivity and mobility of iron derived from soil minerals (24). The reason why the results of the double-layer assay and of the competition in liquid PSBM contradict each other is unknown. Potentially, the experimental conditions in the two assays select for different transcriptional activities in P. orientalis F9. Siderophore production is regulated by Fe^2+^ concentration in the cell (25, 26), while the regulation of safracin production is unknown. Generally, antibiotic production has been shown to be regulated not only via quorum sensing (QS) but also by the presence and absence of interspecies competition (27, 28). Genes coding for proteins with high similarity to the LasI/R and RhlI/R QS systems that regulate the production of multiple virulence factors in P. aeruginosa (29) could not be detected in P. orientalis F9.

Safracin has been shown to be a broad-spectrum antibiotic (30) and, indeed, caused strong inhibition *in vitro*. However, it appears to have a minor role or no role in competition against E. amylovora^Rif^ in detached flowers or against P. ultimum in soil. There are additional metabolites that are major candidates for the antagonistic performance of P. orientalis F9 in the *in vivo* assays. The strain also harbors poaeamide (BOP93_16455) and obafluorin synthesis genes on its genome. Poaeamide inhibited mycelial growth of Rhizoctonia solani and different oomycetes, including P. ultimum (31). This might explain why the phenazine deletion mutant of F9 is still able to antagonize P. ultimum in the cress assay. The β-lactone antibiotic obafluorin produced by P. fluorescens ATCC 39502 showed weak antibacterial activity against a range of bacteria by disk diffusion (32). In addition, P. orientalis F9 also harbors genes with homology to the Hcp secretion island 1-encoded type VI secretion system (H-T6SS) (BOP93_RS26545 to BOP93_RS26640). In the case of Serratia marcescens Db10, the T6SS exhibits antibacterial killing activity (33). Future analyses have to determine if these features of P. orientalis F9 are major players in the strain’s repertoire of antagonistic traits in the apple blossom or cress assay.

Results presented in this paper demonstrate that competition and antagonism are multifactorial and not solely dependent on antibiotic and/or siderophore production. Investigating the causal reason for the different results is, however, out of the scope of this study. The actual cause for antagonism is thus still unclear and could be mediated by the plant host, resource competition, or antibiotics that have not been studied (28, 34, 35). Our results highlight the importance of a proper choice of screening systems that need to be sufficiently close to environmental conditions. Results of *in vitro* screens do not reflect *in situ* results and need to be supplemented by additional corroborative assays.

## MATERIALS AND METHODS

### Cultivation of bacterial strains used.

Bacterial overnight cultures were grown at 26°C in tryptic soy broth (TSB; Oxoid, Karlsruhe, Germany) or King’s B (KB) medium (36). Partial stigma-based medium (PSBM) (14) was also used. Microorganisms used in the study are listed in Table 1. Where appropriate, medium was supplemented with kanamycin or rifampin at a concentration of 40 mg liter^−1^ or 100 mg liter^−1^, respectively.

### Transposon mutagenesis.

Random insertion Tn*10* transposon mutagenesis was performed using pJA1, an oriR6K-based suicide vector. The plasmid contains the Tn*10* transposase and confers kanamycin resistance (37). Donor Escherichia coli SM10 λpir(pJA1) and recipient P. orientalis F9 were grown overnight in either LB medium (10 g liter^−1^ tryptone, 5 g liter^−1^ yeast extract, 10 g liter^−1^ NaCl, pH 7.0 ± 0.2 [Carl Roth]) supplemented with kanamycin or KB medium, respectively. The overnight culture of E. coli SM10 λpir(pJA1) was inoculated into fresh LB medium containing kanamycin and grown to mid-exponential phase. One milliliter of the donor and the recipient in stationary phase was mixed and centrifuged for 30 s at 10,000 × *g*. The bacterial pellet was washed twice with 1× phosphate-buffered saline (PBS) (2.5 g liter^−1^ K_2_HPO_4_, 1.2 g liter^−1^ KH_2_PO_4_) and subsequently spotted onto the center of an isopropyl-β-d-thiogalactopyranoside (IPTG)-containing LB plate (10 μl of a 100 mM IPTG solution, spotted onto the center of the plate). The conjugation plate was incubated at 37°C for 3 to 5 h. A dilution series was plated onto MM2-agar plates (4 g liter^−1^ l-asparagine, 2 g liter^−1^ K_2_HPO_4_, 0.2 g liter^−1^ MgSO_4_, 3 g liter^−1^ NaCl, 10 g liter^−1^ sorbitol, 15 g liter^−1^ agar) supplemented with kanamycin. After 2 to 3 days of growth at 26°C, single colonies were picked and inoculated in 96-well plates containing KB medium and grown for 2 days at 26°C. P. orientalis F9 mutants were screened for two phenotypes: (i) a lack of fluorescence in the iron-limited KB agar and (ii) a lack of growth deficiency halos on KB plates overlaid with E. amylovora^Rif^ in the double-layer assay (see below).

### Identification of transposon insertion sides.

Transposon insertion sites were identified according to Holenstein (38). As previously described (39), transposon insertion-specific primers arPCR-T7 (5′-GCACCTAACCGCTAGCACGTATACGACTC-3′) and ARB6 (5′-GGCCACGCGTCGACTAGTACNNNNNNNNNNACGCC-3′) were used for a primary PCR. In a second, nested PCR, primers arPCR-T7 inner (5′-TGAACGGTAGCATCTTGACGAC-3′) and ARB2 (5′-GGCCACGCGTCGACTAGTAC-3′) were used. A crude DNA extract of selected transposon mutants was prepared by suspending 24- to 48-h-old bacterial colonies in 300 μl of double-distilled H_2_O and incubating them at 95°C for 30 min, followed by centrifugation at 12,000 × *g* for 1 min. The supernatant was diluted 1:10 with double-distilled H_2_O and used as a PCR template. Amplifications were performed using Hotstar *Taq* polymerase (Qiagen). PCR products were sequenced using an ABI Prism BigDye Terminator, version 1.1, cycle sequencing kit (Applied Biosystems) and analyzed using nucleotide NCBI (National Center for Biotechnology Information) BLAST against the P. orientalis F9 whole-genome sequence (1) and protein BLAST using the UniProt database with default settings (40).

### Site-directed mutagenesis of P. orientalis F9.

A phenazine mutant of P. orientalis F9 (Δphen::Kan^r^) was generated by site-directed mutagenesis. For this purpose, the BglII, EcoRI, and EcoRV cut sites within the phenazine gene cluster (Fig. 1) were used. Two fragments of ca. 1.4 kb (primer PhenaF, 5′-GTCGTGGAAGCTGGACAGTG-3′; primer PhenaEVr, 5′-GACTCGGCGATCCTGATTCG-3′; annealing temperature of 58°C) and 1.8 kb (primer PhenaEVf, 5′-TGGTGTTGCCGTGCATCGGG-3′; primer PhenaR, 5′-AACCGGTGAACCCCTTGTTT-3′; annealing temperature of 58°C) of a 5.2-kb BglII/EcoRI fragment of the phenazine gene cluster were amplified by PCR. The 1.4-kb PCR product was digested with BglII/EcoRV, and the 1.8-kb product was digested with EcoRV/EcoRI. The BglII/EcoRV product was ligated in a first step into the similarly digested suicide vector pKAS32 (41), followed by ligation with the EcoRV/EcoRI-digested 1.8-kb fragment and the appropriate cut vector of the first ligation step. The resulting plasmid pSVI1 + 2 harbored the 5′ and 3′ end flanking regions of the BglII/EcoRI phenazine fragment with a 2.2-kb deletion in between. pSVI1 + 2 was EcoRV digested and ligated with a HindII-cut kanamycin cassette from pSB315 (42), resulting in pSVKan. When pKAS32 derivatives are used for the positive selection of a double-allelic exchange, a streptomycin-resistant parental strain is required (41). Spontaneous streptomycin-resistant colonies of P. orientalis F9 were isolated by increasing streptomycin concentrations (starting concentration of 10 μg/ml; final concentration of 100 μg/ml) in KB medium. pSVKan was conjugated into P. orientalis F9 (Sm^r^) using two-parental mating employing E. coli S17-1 λpir (43). Kanamycin- and streptomycin-resistant mutants were selected on MM2 medium containing 40 μg/ml kanamycin and 500 μg/ml streptomycin. Strain P. orientalis F9 (Δphen::Kan^r^ Sm^r^) was tested via PCR using primers for the presence of the kanamycin resistance gene (aph157, 5′-GTCACCGAGGCAGTTCCA-3′; aph606, 5′-CGACCATCAAGCATTTTATC-3′; annealing temperature of 58°C) and primers set before the EcoRV restriction site (DelF, 5′-AGGTGAACGTGTCTTCGGCG-3′; DelR, 5′-CTCCCGATCATGTGATCCGC-3′). DelF and DelR amplified a ca. 700-bp fragment and the integrated kanamycin resistance cassette. The position of the kanamycin cassette within the former EcoRV cutting site was confirmed by sequencing.

### Bacterial growth rate analysis.

For growth rate analysis, a Bioscreen C (Oy Growth Curves Ab, Ltd., Helsinki, Finland) automatic microbiology growth curve analysis system was used. Nine hundred millimeters of medium (KB or PSBM) was pipetted into a reaction mixture, 200 μl of which was not inoculated but used as a negative control for the corresponding growth curves. The remaining 700 μl was inoculated with 3 μl of overnight culture of the tested strain. Three replicates of the inoculated medium, each 200 μl, were loaded in wells of a Bioscreen C honeycomb plate. The plates were incubated at 26°C for 24 h and were shaken every 20 min for 10 s before the absorbance at an optical density of 600 nm (OD_600_) was determined. Growth experiments were performed in two independent trials.

### Siderophore assay.

Siderophore production was tested using chrome azurol S (CAS) agar (44). Bacteria were grown overnight on TSB plates, harvested, and resuspended in PBS to an OD_600_ of 1. Five microliters of the bacterial suspension was spotted onto chrome azurol S agar and incubated for 3 days at 26°C. Siderophore-producing strains induce a color change from a green-blue CAS-iron complex to orange desferrated CAS.

### Phytotoxicity test of P. orientalis F9 and mutant strains.

For the phytotoxicity test, P. orientalis F9 and mutant strains were cultivated overnight on TSB plates. Colonies were resuspended in 1× PBS and adjusted to an OD_600_ of 1. Twenty microliters of a 10^−2^ dilution of the suspension was applied onto the hypanthium of apple flowers. Inoculated flowers were incubated at 26°C in closed boxes with water-saturated paper towels, and necrosis was evaluated 4 days postinfection (dpi).

### Growth inhibition test using a double-layer assay.

For a double-layer assay, the strains were cultivated overnight on KB plates. Colonies were resuspended in 1× PBS and adjusted to an OD_600_ of 1. For bacteria seeded in the top layer, approximately 5 × 10^8^ bacteria were added to 10 ml of 0.75% KB top agar (cooled to 45°C). Fifteen milliliters of a top agar was poured on top of a 12 cm by 12 cm KB agar plate. Ten microliters of each P. orientalis F9 mutant strain tested as an antagonist was spotted onto the solidified top layer surfaces. Growth halos were detected after 2 days of incubation at 26°C.

### Stigma-based medium *in vitro* competition assay.

E. amylovora^Rif^, P. orientalis F9, and mutants were cultivated on KB plates overnight. Colonies were harvested and resuspended in 1× PBS to an OD_600_ of 1 before being serially diluted up to 10^−4^. Twenty microliters of this bacterial suspension was then added to 980 μl of 1× PBS, or, in the case of a coinoculation, 20 μl of each strain was added to 960 μl of 1× PBS. Sixty microliters of the bacterial suspension was directly pipetted into 1,440 μl of PSBM, vortexed, and then aliquoted to 400 μl in three columns of a 96-deep-well plate. After 3 days at 26°C, the CFU counts of E. amylovora^Rif^ were determined. A serial dilution of the inoculated medium was performed up to 10^−7^. Three microliters of each dilution was spotted onto TSB plates supplemented with rifampin (100 μg/ml). Experiments were repeated three times independently.

### Cress assay.

For the cress assay (45), two 1-cm-diameter discs of Pythium ultimum culture grown on malt extract agar (Oxoid) were punched out, laid on the bottom of a 9-cm-diameter petri dish, and carefully overlaid with 14 g of doubly autoclaved soil. P. orientalis F9 and F9 mutants were grown on KB plates overnight and resuspended in 1× PBS to an OD_600_ of 0.1. Ten milliliters of the bacterial suspensions or mock control (PBS only) was evenly spread over the soil surface, followed by 0.4 g of cress seeds (Lepidium sativum). Petri dishes were incubated at 22°C and 65% humidity. After 2, 4, and 6 days of incubation, 20 ml of autoclaved water was added. After 7 days of incubation, the complete above-ground cress biomass was harvested, and its wet weight was determined. Each treatment was performed in duplicates for inoculation of cress only with P. orientalis F9, TM16, TM18, P. orientalis F9 (Δphen::Kan^r^), and P. protegens CHA0 or in quadruplicates for P. ultimum controls and coinoculations of P. orientalis F9, TM16, TM18, P. orientalis F9 (Δphen::Kan^r^), and P. protegens CHA0 with P. ultimum. The experiments were repeated two to three times independently.

### Detached-flower assay.

For the detached-flower assay (46), freshly opened flowers of 2-year-old potted Malus domestica Golden Delicious in the greenhouse were used. E. amylovora^Rif^, P. orientalis F9, and mutants were cultivated and resuspended to an OD_600_ of 1 as described above. A serial dilution of each bacterial suspension was performed. For the E. amylovora control, 20 μl of the 10^−4^ dilution was transferred into 980 μl of PBS; for coinoculations, 20 μl of each strain was transferred into 960 μl of PBS. Twenty microliters of the bacterial suspensions was directly pipetted onto the hypanthium of individual flowers. Mock treatments were performed with 1× PBS. After inoculation, flowers were incubated at 26°C in closed boxes with water-saturated paper towels on the bottom. Two and four days postinfection (dpi), the CFU counts of E. amylovora^Rif^ in eight individual flowers of each treatment were determined as described previously (1). Briefly, petals, pedestals, stamens, and stigmas of the flowers were removed, and the remaining flowers were shaken in 1 ml of 1× PBS for 30 min at 1,400 rpm. Afterwards, tubes were vortexed for 30 s. A serial dilution of the supernatant suspension was performed up to 10^−7^, and 3 μl of each dilution was spotted onto TSB plates supplemented with rifampin (100 μg/ml). Detached-flower assays for transposon mutants TM16 and TM18 (and alternative mutants TM10, TM13, and TM19) were performed during the spring and summer of 2018, and for the phenazine mutant P. orientalis F9 (Δphen::Kan^r^), they were performed in the spring of 2019. Assays were repeated at least three times independently.

### Genome analysis of P. orientalis F9.

The genome of P. orientalis F9 (CP018049.1) was analyzed for additional metabolites and factors with potential antagonistic activity using the Virulence Factors Database (VFDB; virulence factors of bacterial pathogens) and antiSMASH. In addition, blastn/tblastn (NCBI) comparison of the P. orientalis F9 genome and synthesis genes for additional secondary siderophores of pseudomonads was also performed using the sequences for enantio-pyochelin (47), pseudomonine (48), pyridine-2,6-bis(monothiocarboxylic acid) (PDTC) (49), and thioquinolobactin (50) synthesis genes.

## Supplementary Material

Supplemental file 1
