# Supplementary material for: Pseudomonas orientalis F9 Pyoverdine, Safracin, and Phenazine Mutants Remain Effective Antagonists against Erwinia amylovora in Apple Flowers
Source: Appl Environ Microbiol. 2020 Apr 1;86(8):e02620-19. doi: 10.1128/AEM.02620-19 (PMC7117935; doi:10.1128/AEM.02620-19)
Supplement: Supplemental file 1 [file AEM.02620-19-s0001.pdf]

Supplemental material

***Pseudomonas orientalis* F9 pyoverdine, safracin and phenazine mutants remain effective antagonists against *Erwinia amylovora* in apple flowers**

Amanda Santos Kron, Veronika Zengerer, Marco Bieri, Vera Dreyfuss, Tanja Sostizzo, Michael Schmid, Matthias Lutz, Mitja N. P. Remus-Emsermann, and Cosima Pelludat

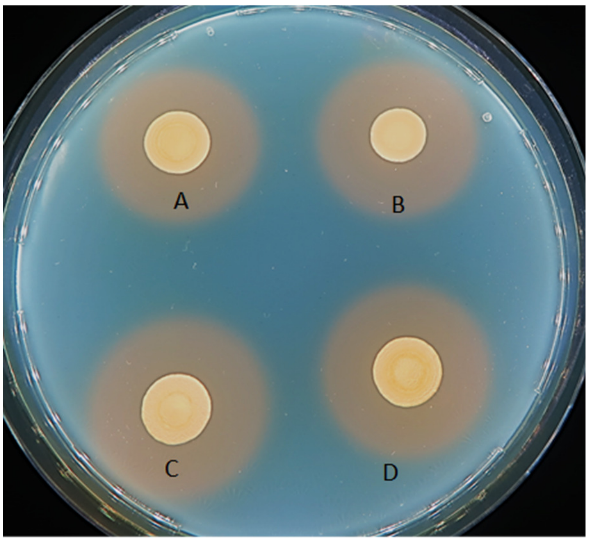

Supplemental Fig. S1:

Siderophore synthesis tested on Chrome azurol S (CAS) agar.

Five µl of a bacterial suspension of *P. orientalis* F9 (A), *P. orientalis* F9Δphen::Kan^R(B), Transposon mutants TM16 (C), and TM18 (D) were spotted onto the agar and incubated for two days at 26°C. Siderophore-production is indicated by an orange halo.

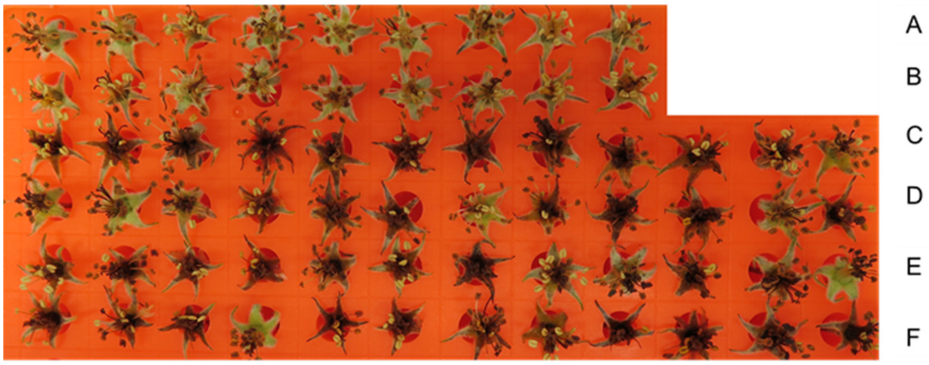

Supplemental Fig. S2:

Phytotoxicity test: nine to twelve freshly opened apple flowers were inoculated with 20 µl of a bacterial suspension. Four-days post infection (d.p.i.) phytotoxicity (necrosis of calyx) of the strains was evaluated.

A: PBS buffer; B: *Pantoea vagans* C9-1 (*E. amylovora* antagonist);

C: *P. orientalis* F9; D: *P. orientalis* F9Δphen::Kan^R; E: *P. orientalis* TM16;

F: *P. orientalis* TM18.
